# Biocide, antifungal susceptibility and virulence characteristics of Clade 1 *Candidozyma auris* strains

**DOI:** 10.1186/s12941-025-00821-8

**Published:** 2025-09-30

**Authors:** Ayşe Kalkanci, Sidre Erganis, Elif Ayça Sahin, Esra Kilic, Sena Algin, Halil Furkan Martli, Beyza Yavuz, Alper Dogan, Fusun Kirca, Sema Turan Uzuntas, Ayşe Çagatan Seyer, Mubarek Taiwo Mustapha, Abdullahi Garba Usman, Meliz Yuvali, Cagri Ergin, Bedia Dinc, Dilber Uzun Ozsahin

**Affiliations:** 1 Faculty of Medicine, Department of Medical Microbiology, Gazi University, Ankara, Turkey; 2 Clinics of Microbiology, Bilkent City Hospital, Ankara, Turkey; 3 DESAM Research Institute, Near East University, Nicosia, Cyprus; 4 Near East University, Operational Research Centre in Healthcare, Nicosia, Cyprus; 5 Cyprus Faculty of Medicine, Department of Biostatistics, Near East University, Nicosia, Cyprus; 6 Faculty of Medicine, Department of Medical Microbiology, Pamukkale University, Denizli, Turkey; 7 Department of Medical Diagnostic Imaging, College of Health Sciences, University of Sharjah, Sharjah, United Arab Emirates; 8 Research Institute for Medical and Health Sciences, University of Sharjah, Sharjah, UAE

**Keywords:** *Candidozyma (Candida*) *auris*, Clade 1, Biocide, Antifungal, Virulence

## Abstract

**Background:**

*Candidozyma auris* is an emerging multidrug-resistant fungal pathogen responsible for nosocomial outbreaks worldwide. In addition to antifungal resistance, its ability to persist in the hospital environment and tolerate commonly used biocides presents a critical challenge for infection control. However, the relationship between biocide tolerance, antifungal resistance, and virulence traits in *C. auris* remains poorly understood.

**Methods:**

In this study, 47 *C. auris* Clade 1 isolates were evaluated using phenotypic and genotypic methods. ITS region sequencing was performed using Oxford Nanopore technology. Susceptibility testing was conducted for seven antifungal agents and four biocides using the CLSI reference microdilution method. Virulence factors including biofilm formation, secreted aspartyl proteinase, esterase, caseinase, phospholipase, and hemolysis were assessed.

**Results:**

All isolates were identified as Clade 1. MIC values for antifungals ranged from 0.015 to 64 µg/mL, and for biocides from 0.0078 to 128 mg/L. Fluconazole resistance was found in 31% of isolates, while amphotericin B resistance was 4%; no echinocandin resistance was observed. Biofilm and SAP activity were detected in all isolates; esterase was positive in 87%, and caseinase in 4%. Statistically significant correlations were observed between amphotericin B and isavuconazole MICs (ρ = 0.32, p = 0.028), amphotericin B and triclosan MICs (ρ = 0.35, p = 0.018), and amphotericin B MICs and caseinase activity (ρ = 0.31, p = 0.035). These findings suggest potential phenotypic links between antifungal resistance and specific virulence traits.

**Conclusions:**

This is the first study from Türkiye to evaluate the antifungal and biocide susceptibility alongside virulence characteristics of Clade 1 *C. auris*. While statistically significant associations were observed, we acknowledge that resistance mechanisms and pathogenicity pathways are distinct. Therefore, these correlations should be interpreted cautiously and warrant further investigation at the molecular level.

**Supplementary Information:**

The online version contains supplementary material available at 10.1186/s12941-025-00821-8.

## Introduction

*Candidozyma auris* (formerly *Candida auris*) is a multidrug-resistant yeast that has rapidly emerged as a significant cause of health care-associated infections (HAIs) worldwide [1]. Its ability to persist in hospital environments, colonize skin and surfaces, and resist multiple classes of antifungal agents has led to frequent outbreaks and treatment failures, particularly in intensive care settings. In recognition of its global health threat, the World Health Organization (WHO) classified *C. auris* as a “critical priority fungal pathogen” in 2022 [2–4]. One of the major concerns surrounding *C. auris* is its simultaneous resistance to both antifungal agents and hospital-grade disinfectants [5–7]. In clinical settings, disinfectants such as quaternary ammonium compounds (QACs), chlorhexidine, and sodium hypochlorite are widely used to eliminate surface contamination. However, several studies have demonstrated that *C. auris* strains can tolerate or survive these agents under certain conditions, particularly when organized within biofilms [8–10]. Biofilm formation is not only associated with environmental persistence but also with increased resistance to antifungals and host immune defenses.

Despite increasing reports on antifungal resistance and virulence traits, few studies have simultaneously evaluated the relationship between biocide susceptibility, antifungal resistance, and virulence profiles in *C. auris* isolates [11, 12]. The antifungal susceptibility profile of *C. auris* is particularly alarming because of its resistance to multiple classes of antifungal agents. Globally, approximately 90% of isolates are resistant to fluconazole, 30% are resistant to amphotericin B, and approximately 5% are resistant to echinocandins [13, 14].

Additionally, most published studies have been limited to Clade II or IV isolates, while Clade I remains the most widespread and clinically relevant lineage globally. The lack of integrated data from Türkiye further complicates local infection control strategies. By integrating phenotypic susceptibility profiles with virulence assessments, we sought to identify potential correlations between resistance and pathogenicity features and contribute to the growing understanding of *C. auris* epidemiology and control. Notably, *C. auris* strains show significant variation in antifungal susceptibility depending on their clade. For example, Clades I and IV are often associated with higher resistance rates, whereas Clade II isolates are generally more susceptible to antifungal agents [15]. The species name *Candida auris* was first introduced in a 2009 study by Satoh and Makimura [4], who isolated yeast from the external ear canal of a patient in Japan. In 2024, Liu et al. [16] proposed reclassifying this species into a new genus, renaming it *Candidozyma auris*. The resistance of *C. auris* to various biocidal agents has become increasingly evident. For example, Erganis et al. [17] demonstrated that clinical *C. auris* isolates (Clade I) presented substantial resistance to benzalkonium chloride (BNZ) with only variable susceptibility to chlorine and chlorhexidine (CHX), depending on the concentration and surface conditions.

This study aimed to evaluate the antifungal susceptibility, biocide tolerance, and virulence characteristics of The new fungal threat *C. auris* [18] 47 clinical *Candidozyma auris* Clade 1 isolates collected from multiple centers in Türkiye.

## Materials and methods

### Candidozyma (Candida) auris isolates

A total of 47 clinical *C. auris* isolates (20 blood, 12 urine, and 15 skin) that were phenotypically identified at the species level by matrix-assisted laser desorption/ionization (MALDI) time-of-flight (TOF) (Vitek MS™, bioMerieux) were reidentified genotypically. The quality control strain MBL *Candida auris* CDC B11903 was used.

### Molecular identification

Lysozyme was added to *C. auris* lysis buffer (250 mM NaCl, 100 mM EDTA (pH = 8), 1% SDS, and 8 M guanidine thiocyanate), and digestion buffer (10 mM Tris (pH 10.5), 1 mM EDTA, and 0.15 mM NaCl) and 10 μL of proteinase K were used for DNA isolation from the yeast suspensions. The supernatant was purified using an equal volume of phenol‒chloroform–isoamyl alcohol (25:24:1). Next-generation sequencing (NGS) was performed on the genomic DNA samples. The Ligation Sequencing Kit (SQK-LSK109; Oxford Nanopore Technologies) and the Expansion Kit (EXP-NBD114) were used for genome library preparation and barcoding. The genome libraries were loaded onto a MinION™ FLO-MIN106 flow cell (Oxford Nanopore Technologies). Following sequencing, the data were obtained in FAST5 format and converted to FASTQ format. The resulting reads were subsequently transferred to Geneious Prime 2023.2.1 software. For the nanopore read configuration, sequences were aligned using de novo assembly. To find the best species match, local BLAST version 2.12.0 + was used. Reference genomes for *Candida auris* (strain B11220) were downloaded from NCBI.

### Antifungal susceptibility results

All the isolates were tested for susceptibility to amphotericin B, fluconazole, voriconazole, itraconazole, posaconazole, caspofungin, and isavuconazole using the CLSI M27-A2 reference broth microdilution method [19]. The obtained MIC values were recorded. The drug concentration ranges used were 0.015–8 µg/mL for echinocandins, 0.03–16 µg/mL for polyenes and 0.125–64 µg/mL for azoles. For azoles and echinocandins, the MIC was defined as the lowest concentration resulting in 50% inhibition, whereas for polyenes, the MIC was defined as the lowest concentration resulting in 100% inhibition. For the MIC study, *Candida parapsilosis* ATCC 22019 and *Candida krusei* ATCC 6258 were used as quality control strains. As planned, the quality control strain MBL *Candida auris* CDC B11903 was purchased and included in the study. Breakpoints for *C. auris* were proposed by the Centers for Disease Control and Prevention (CDC). The strains were evaluated accordingly. MIC values of 2 µg/mL for amphotericin B, 32 µg/mL for fluconazole, and 4 µg/mL for caspofungin and anidulafungin were accepted as resistance limit values [20].

### Biocide susceptibility

The biocides benzalkonium chloride (BNZ), chlorhexidine digluconate (CHX), triclosan (TRC), and sodium hypochlorite (SHC) were used in this study. Sensitivity tests for BNZ (0.0625–128 mg/L), CHX (0.0625–32 mg/L), TRC (0.0078–8 mg/L), and SHC (0.0078–8 mg/L) were adapted from the CLSI M27-A2 reference microdilution method. The MIC values for these chemicals were determined using the broth microdilution method. Epidemiological cut-off values (ECOFFs) were calculated specifically for *C. auris* in relation to biocide susceptibility determined on the basis of *C. albicans* literature previously reported [21]. The MIC for each biocide was defined as the lowest concentration resulting in ≥ 50% inhibition of visible growth compared to the drug-free control well, as determined by visual inspection. This approach is consistent with the CLSI M27-A3 method used for antifungals and with previously published biocide susceptibility studies involving *C. auris*. Although some biocidal agents may have fungicidal properties, MIC-80, MIC-100, or MFC values were not determined, as there are currently no standardized or validated reference methods for defining these thresholds in biocide testing for yeasts. The primary aim of this study was to assess the relative inhibition profile across isolates, rather than to determine definitive killing endpoints.

### Virulence tests

The following virulence factors were investigated: biofilm formation, proteinase (SAP), caseinase, phospholipase, esterase, and haemolytic activity.

#### Biofilm formation

The biofilm formation ability was also tested in both tubes and polystyrene microplates. For this purpose, *C. auris* isolates were incubated overnight at 37 °C on SDA and passed into yeast peptone dextrose (YPD) broth, followed by overnight incubation at 30 °C in a shaking incubator. From the resulting planktonic cells in the logarithmic growth phase, a suspension was prepared in RPMI 1640 medium at a concentration of 10^6^ CFU/mL. A volume of 200 µL of the prepared suspension was transferred to 96-well microplates and incubated at 37 °C for 24 h. After incubation, the plates were washed three times with PBS, and the biofilm biomass was assessed using the crystal violet assay, whereas the metabolic activity of viable microorganisms was tested using the MTT reduction assay [22, 23]. Each isolate was tested in at least 3 wells per experiment, and the experiment was repeated on three different days. The results were compared to those of *Candida albicans* MYA 274, a biofilm-positive control strain. Classification: Biofilm production was scored as:

- 0 = none (OD ≤ negative control),
- 1 = weak (OD ≤ 2 × control),
- 2 = moderate (2–4 × control),
- 3 = strong (> 4 × control).

#### Hemolytic activity

Haemolytic activity was assessed by culturing the isolates on SDA supplemented with 7% sheep blood. SDA supplemented with 7% defibrinated sheep blood. Assay: 10 µL inoculum was spotted onto plates and incubated for 72 h. Result:

- Greenish halo = alpha hemolysis (positive),
- Clear halo = beta hemolysis,
- No change = negative.

#### Secreted aspartyl proteinase (SAP) activity

To demonstrate the presence of SAP, the development of a clear lysis zone around the isolates on bovine serum albumin agar (SSAA) was evaluated. Assay: 10 µL of standardized yeast suspension (~ 10⁷ CFU/mL) was spotted onto plates and incubated for 5 days. Interpretation: A clear zone around colonies indicated proteinase activity.

- 0 = no halo,
- 1 = halo diameter < colony diameter (weak),
- 2 = halo ≈ colony (moderate),
- 3 = halo > colony (strong).

#### Phospholipase activity

The presence of phospholipase enzymes was investigated by observing the formation of a precipitation zone on egg yolk agar (EYA). Sabouraud dextrose agar supplemented with 1 M NaCl, 0.005 M CaCl₂, and 8% sterile egg yolk emulsion. Assay: 5 µL inoculum was placed centrally and incubated for 5 days.

Scoring: A precipitation zone around the colony indicated activity.

- Pz = colony diameter/(colony + zone diameter)
- Pz = 1.0 = no activity, 0.89–0.99 = weak, 0.70–0.89 = moderate, < 0.70 = strong.

#### Esterase activity

For esterase production, the appearance of a precipitation zone on Tween 80 agar was examined. Tween 80 agar containing peptone (10 g/L), NaCl (5 g/L), CaCl₂ (0.1 g/L), and 1% Tween 80. Assay: 10 µL inoculum was placed onto the surface and incubated for 3 days. Evaluation: White precipitate (calcium oleate) around colonies = positive esterase activity. Scored as positive or negative.

#### Caseinase activity

Casein hydrolysis was assessed using skim milk agar. Skim milk powder (10%, w/v) in nutrient agar base (Oxoid). Assay: 10 µL of inoculum was spotted and incubated for 48 h. Result: Clear halo = positive caseinase activity; absence of halo = negative. Only presence/absence was recorded.

References [24, 25] were followed for in vitro virulence testing.

### Statistical analysis

Descriptive statistics, including minimum inhibitory concentration (MIC) ranges, MIC_50_, MIC_90_, and ECOFF values, were used to summarize antifungal and biocide susceptibility profiles. The Mann–Whitney U test was used to compare the MICs of antifungal agents between isolates. The Kruskal‒Wallis test was performed to determine whether there were statistically significant differences in the triclosan MIC values among *C. auris* isolates with varying levels of biofilm production. Correlations between biofilm formation strength, antifungal resistance (particularly fluconazole and voriconazole MICs), and esterase activity were evaluated using Spearman’s rank correlation coefficient (Spearman’s ρ). A heatmap was generated using z-score normalization (mean-centred and unit-variance scaled) of all the quantitative variables, including antifungal MICs, biocide MICs, and binary virulence factor scores. The standardized data matrix was visualized using the “seaborn.heatmap function” (Python 3.11) to highlight relative phenotypic patterns across isolates. All the statistical analyses were performed using appropriate nonparametric methods due to the nonnormal distribution of the MIC data. A *p* value of < 0.05 was considered to indicate statistical significance.

Hierarchical clustering was performed to assess the overall phenotypic similarity among *C. auris* isolates on the basis of antifungal MICs, biocide MICs, and virulence characteristics. Prior to clustering, all numerical variables were standardized using z-score normalization (mean = 0, standard deviation = 1). The combined data matrix included antifungal and biocide MIC values as well as binary virulence traits such as esterase, phospholipase, caseinase, and biofilm production. A distance matrix was computed using the Euclidean distance, and clustering was conducted by Ward’s linkage method, which minimizes the total within-cluster variance. The resulting dendrogram was generated using the “scipy.cluster.hierarchy” module in Python.

## Results

A number was taken from GenBank for each of the seven chromosomes of clade 1 isolates. The GenBank accession numbers of clade 1 isolates are CP147431–CP147437 for sample 1, CP147438–CP147444 for sample 2, CP147445–CP147451 for sample 3, CP147452–CP147458 for sample 4, CP147459–CP147465 for sample 5, CP147466–CP147472 for sample 6, CP147473–CP147479 for sample 7, and CP147480–CP147486 for sample 8.

Among the 47 *C. auris* Clade I isolates studied, antifungal susceptibility testing revealed consistently high MICs for fluconazole (MIC_50_: 16 µg/mL, MIC_90_: 32 µg/mL). Resistance to fluconazole was nearly universal. The MIC values of amphotericin B were moderately elevated (MIC_50_: 0.5 µg/mL, MIC_90_: 1 µg/mL), whereas caspofungin had low MICs (MIC_50_: 0.12 µg/mL, MIC_90_: 0.25 µg/mL), indicating preserved susceptibility. No statistically significant differences were noted for amphotericin B or echinocandin MICs between different isolation sites (*p* > 0.05). Table 1 shows the results. The supplementary file shows all MICs and virulence results.

**Table 1:** MIC_50_, MIC_90_ and ECOFF values of antifungal agents against *C. auris*

	MIC_50_	MIC_90_	ECOFF
	(µg/mL)		
AMPHO	0.5	1	2
VORI	2	8	8
FLU	16	32	32
ITRA	1	2	2
POSA	0.25	0.5	0.5
CASPO	0.12	0.25	0.5
ISAVU	0.12	0.5	1

Epidemiological cutoff values (ECOFFs) were not formally calculated in this study due to the limited number of *C. auris* isolates and the absence of an internationally accepted reference method for biocide susceptibility in yeasts. Instead, previously published MIC thresholds derived from *Candida albicans* studies were adopted solely as reference points to indicate potential reductions in susceptibility. These included: 0.25 mg/L for triclosan (TRC), 16 mg/L for benzalkonium chloride (BNZ), 0.5 mg/L for chlorhexidine (CHX), and 0.03 mg/L for sodium hypochlorite (SHC) [21]. We stress that these values are not official ECOFFs for *C. auris* and were used only for epidemiological comparison, not for definitive resistance classification. Table 2 shows the MIC_50_, MIC_90_ and ECOFF values for the four biocides.

**Table 2:** MIC_50_, MIC_90_ and ECOFF values of biocides against *C. auris*

	MIC_50_	MIC_90_	ECOFF
	(mg/L)		
TRC	0.25	0.25	0. 5
BNZ	16	16	32
CHX	0.5	0.5	2
SHC	0.01	0.02	0.05

### Virulence factor results

1. *Biofilm formation*

All isolates formed biofilms, with 95% classified as strong producers based on the MTT assay (OD > 4 × negative control). Quantitative absorbance values ranged from 0.32 to 1.12 at 490 nm. *Candida albicans* MYA-274 was used as the biofilm-positive control.

2. *Secreted aspartyl proteinase (SAP)*

Proteinase activity was observed in 100% of isolates. Clear lysis zones were present on BSA agar with activity scores ranging from moderate to strong.

3. *Esterase activity*

Esterase production was detected in 41 of 47 isolates (87%), as evidenced by the precipitation zone on Tween 80 agar. The remaining 6 isolates were esterase-negative.

4. *Caseinase activity*

Only 2 isolates (4%) exhibited caseinase activity, producing minimal hydrolysis halos on skim milk agar. All other isolates were negative.

5. *Phospholipase activity*

No isolates demonstrated phospholipase activity. Colonies showed no precipitation zones on egg yolk agar after 5 days of incubation.

6. *Hemolytic activity*

All isolates exhibited alpha hemolysis on sheep blood agar, with greenish discoloration around colonies. No beta hemolysis or negative results were recorded.

**Fig. 1:**
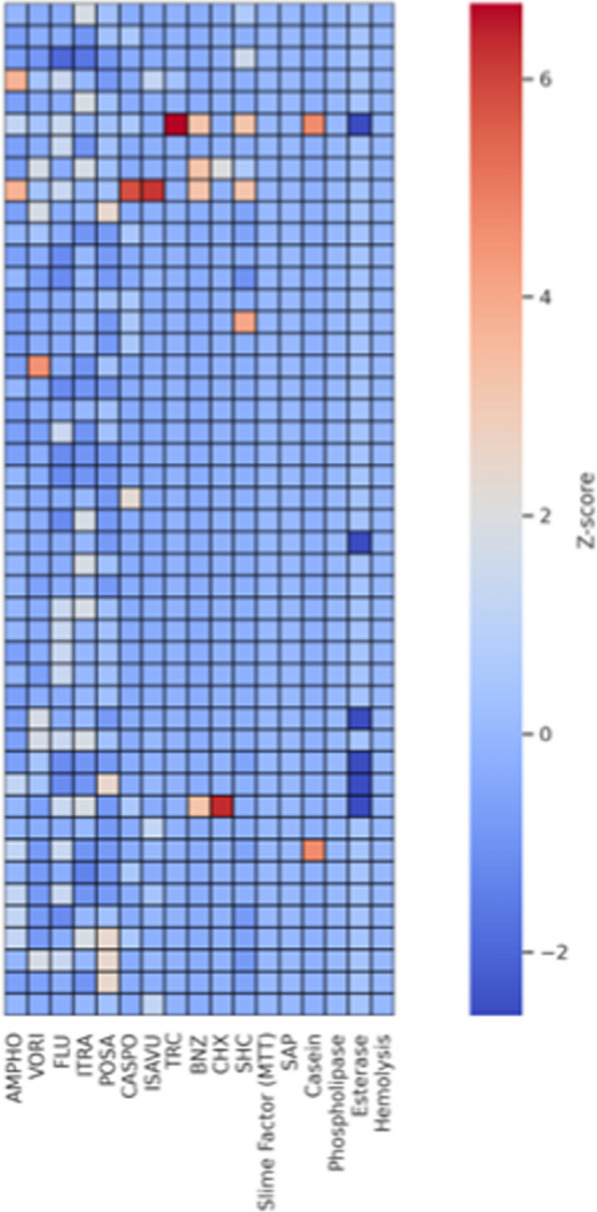
Spearman correlation heatmap of the associations among antifungal MICs, biocide MICs, and virulence factors in *Candidozyma (Candida) auris*. The heatmap displays pairwise Spearman correlation coefficients (r) between quantitative variables. Red tones represent higher-than-average values, whereas blue tones indicate below-average phenotypes. Correlation analysis revealed several statistically significant associations (Spearman ρ > 0.3, *p* < 0.05) between antifungal MIC values, biocide susceptibility, and virulence traits. Note the cross-resistance between amphotericin B and isavuconazole MICs; amphotericin B TRC MICs; caseinase activity; itraconazole and CHX MICs; and caspofungin and BNZ MICs

With respect to virulence, all the isolates exhibited alpha-haemolysis, biofilm and SAP activity. Only 2 isolates were caseinase positive. None of them were phospholipase positive, while 41 of them were esterase positive. Biofilm formation was robust across isolates, with 95% showing strong slime production (+ +  + by MTT assay). This heatmap presents an integrated phenotypic profile of clinical *C. auris* isolates. Higher log_10_ MICs (red shading) imply reduced effectiveness or strong virulence expression, whereas lower values (blue) reflect higher sensitivity or weak/no expression. Correlation analysis revealed several statistically significant associations (Spearman ρ > 0.3, p < 0.05) between antifungal MIC values, biocide susceptibility, and virulence traits. Moderate positive cross-resistance was observed between amphotericin B and isavuconazole MICs (ρ = 0.32, p = 0.028), suggesting a potential shared resistance mechanism or coselection among isolates. Amphotericin B also showed cross-resistance, with MIC values for TRC (ρ = 0.35, p = 0.018) and caseinase activity (ρ = 0.31, p = 0.035), indicating that resistance to this polyene might be linked to both reduced biocide susceptibility and increased proteolytic virulence. Additionally, itraconazole MICs were positively correlated with chlorhexidine MICs (ρ = 0.32, p = 0.030), whereas caspofungin MICs were significantly correlated with BNZ MICs (ρ = 0.39, p = 0.007). Figure 1 shows a Spearman correlation heatmap showing the associations among antifungal MICs, biocide MICs, and virulence factors in *C. auris*. These findings suggest that resistance patterns between certain antifungals and biocides may overlap and could be influenced by common resistance determinants or membrane adaptation mechanisms. Further mechanistic studies are warranted to validate these associations and explore their clinical implications.

**Fig. 2:**
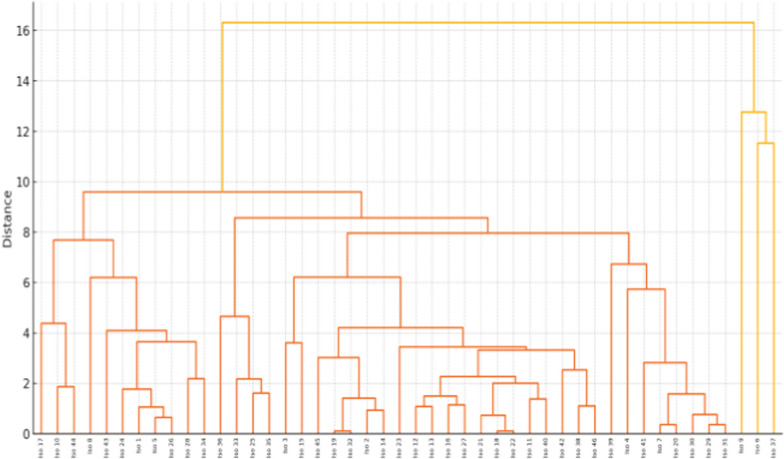
Hierarchical clustering dendrogram of *C. auris* isolates on the basis of their antifungal MICs, biocide MICs, and virulence profiles. The analysis was performed using Ward’s linkage method on standardized (z-score) phenotypic data. The clustering illustrates isolate-level phenotypic similarity, potentially reflecting shared resistance or virulence patterns. Cluster 1 included 43 isolates with high MICs for TRC and frequent biofilm and esterase positivity. Clusters 2 (n = 2) and 3 (n = 1) presented concurrently high TRC and FLU MICs, with variable virulence traits

The isolates were grouped into three primary clusters on the basis of visual inspection and a maximum-cluster threshold (t = 3). The cluster characteristics were summarized by calculating the prevalence of high MIC values (e.g., TRC ≥ 0.25 mg/L, FLU ≥ 32 mg/L) and virulence positivity rates within each cluster. Figure 2 shows a hierarchical clustering dendrogram of *C. auris* isolates on the basis of their antifungal MICs, biocide MICs, and virulence profiles.

*Cluster 1 (n* = *43)*: Isolates predominantly presented high TRC MICs and were frequently positive for biofilm production and esterase activity.

*Cluster 2 (n* = *2)*: Isolates presented MICs with concurrent high TRC and fluconazole (FLU) but lacking esterase activity.

*Cluster 3 (n* = *1)*: This cluster consisted of a single isolate showing simultaneous resistance to TRC and FLU, with positive biofilm and esterase phenotypes.

## Discussion

The interplay among antifungal resistance, biocide tolerance, and virulence expression in *Candidozyma (Candida*) *auris* is a critical challenge for infection control and patient management. In this study, we explored phenotypic patterns across clinical isolates, with a particular focus on biofilm production, esterase activity, and susceptibility to fluconazole and voriconazole. *Candidozyma auris* presents complex resistance phenotypes to both antifungals and biocides, supported by biofilm formation, virulence factor expression, and environmental adaptability. Control of this pathogen requires a multipronged strategy that incorporates targeted biocide use, environmental decontamination, and careful screening of colonized patients. Research into the molecular mechanisms driving resistance, especially within dry-surface biofilms, will enhance our ability to select effective disinfectants. Infection control policies must evolve to include dynamic resistance monitoring and outbreak-tailored decolonization regimens. Only through such integrated strategies can we hope to curb the global spread of this tenacious fungal pathogen [26, 27].

Spearman correlation analysis was conducted to assess potential associations between antifungal susceptibility, biocide tolerance, and virulence phenotypes in *C. auris*. A comprehensive heatmap revealed generally weak correlations across most variables. However, several statistically significant associations emerged with moderate effect sizes (ρ > 0.3, *p* < 0.05). These findings may reflect cross-resistance patterns involving alterations in the fungal cell membrane or efflux-mediated tolerance mechanisms. Interestingly, no strong correlation was found between the MICs of fluconazole and CHX (ρ = 0.12) or between the elevated MICs of fluconazole and the combined biocide (mean ρ = 0.23), contradicting previously reported associations. Similarly, correlations between virulence factors such as biofilms and esterase production with fluconazole or TRC MICs were negligible (ρ < 0.3), indicating that these phenotypes alone are not predictive of antifungal or biocide resistance. Although ECOFF values were applied in this study, it is important to note that they were not derived from our MIC dataset**,** nor do they represent formal *C. auris*-specific wild-type distributions. Instead, thresholds were adapted from *C. albicans-*based literature and previously published *C. auris* studies**,** serving only as indicators of reduced susceptibility trends**.** These values do not reflect resistance mechanisms and should not be interpreted as clinical breakpoints or fungicidal thresholds**.** Future studies with larger isolate numbers and standardized biocide testing protocols are required to establish reliable ECOFFs for *C. auris*. While all *C. auris* isolates in this study presented elevated MICs for BNZ, TRC, and CHX when assessed against *Candida albicans*-based ECOFF thresholds, notably, species-specific clinical breakpoints for *Candidozyma auris* biocide susceptibility have not yet been established. Therefore, these results should be interpreted as indicators of reduced susceptibility rather than definitive resistance. In summary, the analysis suggests that while general cross-resistance trends across antifungals and biocides are limited, certain drug pairs, particularly amphotericin B, TRC, and BNZ, may share susceptibility profiles, potentially driven by common cellular targets or adaptive responses.

*Candidozyma (Candida*) *auris* has emerged as a critical health care-associated pathogen, largely because of its ability to persist in hospital environments and resist both antifungal drugs and common disinfectants. Although we could not conclude any biocide resistance, we previously showed that this species is resistant to several biocides frequently used in health care, particularly quaternary ammonium compounds (QACs), such as BNZ, and chlorhexidine-based solutions [16]. Although these agents are routinely applied for surface or skin decontamination, they often fail to eradicate *C. auris*, especially when they are present within biofilms. Environmental surfaces in hospitals, including medical equipment and patient surroundings, can serve as reservoirs for persistent colonization. Biofilm-forming strains of *C. auris* further increase the difficulty of eradication by reducing the efficacy of standard disinfection practices [28]. This pathogen’s robust ability to colonize dry surfaces has been associated with hospital outbreaks, demonstrating that environmental contamination plays a central role in its transmission [29]. Therefore, understanding biocide tolerance mechanisms and optimizing disinfection strategies are crucial to reducing the spread of *C. auris* in clinical settings.

Biofilm formation is a fundamental virulence strategy of *C. auris*, which not only protects cells from antifungal agents but also contributes significantly to biocide tolerance [30, 31]. Dry surface biofilms have recently been recognized as a particularly concerning phenotype that is capable of persisting despite treatment with sodium hypochlorite [28]. Transcriptomic analyses of these biofilms revealed the upregulation of efflux pumps and iron acquisition systems, contributing to metabolic adaptation and reduced susceptibility. This mechanism parallels the behaviour observed in bacterial biofilms and emphasizes that biofilm-mediated resistance in fungi should be evaluated more rigorously. Clinical isolates have demonstrated planktonic susceptibility but form highly tolerant biofilms on dry abiotic surfaces, particularly under low-nutrient or organic-rich conditions [32]. The biofilm matrix is believed to reduce biocide penetration and promote the survival of embedded cells, especially during intermittent or low-level disinfection. Consequently, surface disinfection protocols must address both planktonic and biofilm forms of *C. auris* [33].

Virulence attributes such as phospholipase production, esterase activity, haemolysis, and an aggregative phenotype may contribute to environmental persistence and biocide tolerance in *C. auris*. Studies have shown that isolates with high biofilm-forming potential often coexpress these virulence factors, which may reinforce the integrity of the biofilm and shield cells from chemical insults [16, 32]. It is plausible that the extracellular matrix produced by strong biofilm-forming strains also retains or neutralizes biocides before they can exert lethal effects. In some models, aggregative phenotypes exhibited both increased biofilm robustness and decreased susceptibility to hydrogen peroxide or CHX [32]. These phenotypic traits could thus serve as biomarkers to predict disinfection outcomes. Further studies correlating specific virulence factors with biocide MIC values will be crucial for refining infection control protocols, especially in outbreak situations. Understanding these links can also help design targeted surface decontamination approaches in high-risk wards.

In a study involving Turkish clinical isolates, all seven tested *C. auris* strains demonstrated moderate to strong biofilm-forming capacity. Interestingly, while biofilm positivity was consistent, the expression of other enzymatic virulence traits, such as proteinase activity and haemolysis, was entirely absent, indicating a strain-specific virulence phenotype. The phenotypic profile of these isolates suggests that biofilm formation may be a dominant and conserved virulence mechanism across different geographic regions [34]. Moreover, the weak activity of phospholipase and esterase among Turkish isolates contrasts with the strong enzyme production commonly observed in *C. albicans*, emphasizing species-specific virulence adaptations. Understanding these phenotypic patterns may help identify more effective treatment approaches and infection control strategies.

The phenotypic variability in *C. auris* virulence traits is further supported by studies that used in vitro assays to assess enzymatic and adhesion properties. Li et al. [30] found that untreated *C. auris* isolates have high levels of adhesion to abiotic surfaces, elevated cell surface hydrophobicity (CSH), and considerable biofilm mass, as determined by XTT and violet crystal assays. Scanning electron microscopy revealed dense, multilayered biofilms with strong surface adhesion, confirming the phenotypic positivity of these virulence traits. The transcriptomic data supported these findings by revealing the downregulation of adhesion- and biofilm-associated genes. Overall, these results show that adhesion, CSH, and biofilm formation are strongly positive phenotypes in most *C. auris* isolates.

Despite the detection of multiple virulence factors, the extent and consistency of their expression vary across *C. auris* strains. As reviewed by Gómez-Gaviria et al. [35], biofilm formation remains the most reliably detected positive trait, whereas phenotypic switching and dimorphism (yeast-to-hyphal or pink‒white transitions) are less prominent but are still documented under specific environmental cues. These phenotypes enable *C. auris* to adapt rapidly to changes in host environments and antifungal pressure. Additionally, secreted exoenzymes such as phospholipase and haemolysin have been inconsistently reported; in some isolates, these activities are undetectable, suggesting partial or clade-specific expression. The presence or absence of these traits may affect tissue invasion potential and immune evasion. These findings underscore the importance of performing comprehensive phenotypic profiling when evaluating strain virulence. Accurate assessment of trait positivity or negativity may assist in epidemiological mapping and inform the design of targeted antifungal or antivirulence therapies.

Several studies have highlighted the variability in the biocidal susceptibility of *C. auris*, which is dependent on the concentration, formulation, and contact time of the agent. For example, while high concentrations of chlorine-based disinfectants achieve acceptable levels of reduction, lower concentrations may not be effective, especially in the presence of organic loading [16, 36]. Similarly, CHX has inconsistent activity, with some studies reporting suboptimal reduction even at concentrations typically used for hand hygiene and skin antisepsis [33]. Octenidine-based solutions are more reliable, showing stronger fungicidal activity within shorter exposure times. The inconsistent susceptibility of *C. auris* strains, particularly across different clades, further complicates infection control strategies, suggesting that disinfectant efficacy testing must be tailored for each institutional setting [16]. This is especially important in high-risk environments such as ICUs, where the potential for nosocomial transmission is heightened by environmental persistence. Regulatory frameworks should thus mandate biocide efficacy validation not only against standard test organisms such as *C. albicans* but also against biofilm-forming *C. auris* isolates [36]. Genomic analyses have indicated that prolonged environmental exposure to subinhibitory concentrations of biocides might select for strains with increased drug tolerance [37, 38]. However, this relationship is not uniform across all strains, and current data do not consistently demonstrate a direct link between antifungal resistance and biocide insensitivity. Nevertheless, the potential for cumulative adaptive responses, especially in clonal strains within outbreak settings, warrants cautious interpretation. Thus, comprehensive surveillance programs should monitor both antifungal and biocide susceptibility profiles concurrently [39].

Persistent environmental contamination by *C. auris* substantially increases the risk of health care-associated infections. This pathogen’s ability to survive on surfaces for up to several weeks, combined with its high transmissibility, positions it among the most environmentally stable fungal agents currently known. Consequently, enhanced cleaning protocols must include validated surface disinfectants that are active against biofilm forms, as well as proper contact times and mechanical cleaning. Additionally, routine environmental screening and decontamination of high-touch surfaces are essential for outbreak control. The implementation of no-touch disinfection methods such as UV-C light or hydrogen peroxide vapour may complement manual cleaning, particularly in patient turnover situations [40, 41].

Another major consideration is the impact of host colonization and skin decontamination protocols on outbreak management. Unlike many *Candida* species that colonize mucosal sites, *C. auris* preferentially colonizes the skin, which may explain its relatively high rates of environmental dissemination [42, 43]. Standard decolonization agents such as 2–4% CHX have shown limited efficacy against skin-colonizing *C. auris*, particularly in its biofilm form [40]. In contrast, povidone–iodine preparations may offer superior efficacy, although patient tolerance and widespread availability may limit their routine use. Newer formulations, such as continuously active disinfectants or antiseptic-impregnated wash mixtures with octenidine, may prove beneficial [33, 42]. A tailored approach to patient decolonization, considering resistance profiles and colonization burden, will be critical for reducing nosocomial transmission.

Haq et al. reported the effectiveness of a novel one-step anionic surfactant disinfectant containing dodecylbenzene sulfonic acid against multiple clades of *C. auris*, including isolates from clades I–IV. Compared with traditional quaternary ammonium compounds, which have shown limited efficacy, this new disinfectant demonstrated promising activity and practical advantages, such as rapid action and minimal residue [44]. Cadnum et al. [45] reported that sporicidal and hydrogen peroxide-based products were significantly more effective against *C. auris* than were quaternary ammonium-based disinfectants. This is particularly concerning given that many health care settings continue to rely on such less effective agents. A review by Omardien and Teska [46] revealed that *C. auris* biofilms, which are commonly formed on skin and surfaces, further complicate disinfection. These biofilms can shield fungal cells from disinfectants such as CHX and hydrogen peroxide, reducing their efficacy. The biofilm matrix can trap antifungal molecules such as fluconazole, rendering them ineffective. In this context, the tolerance of *C. auris* to environmental stressors, including high salinity and desiccation, further enhances its persistence and dissemination in health care facilities. Ku et al. [6] emphasized that although chlorine-based products remain among the most effective for surface disinfection, the addition of mechanical cleaning protocols is often necessary for thorough decontamination. Moreover, the persistent colonization of patients despite body washes with CHX calls into question the long-term efficacy of biocidal strategies alone.

*Candidozyma (Candida) auris* is now recognized as a global fungal threat because of its multidrug resistance and persistence in health care environments. The earliest Japanese isolates, which were derived primarily from noninvasive ear discharge samples, belonged to clade II and presented relatively low resistance rates; only 20% were resistant to fluconazole, while all were susceptible to echinocandins and amphotericin B [47]. These findings suggest that clade II strains, which are common in East Asia, may inherently carry lower antifungal resistance burdens. However, surveillance is still essential, as clade shifts or horizontal transmission could introduce resistance elements. In Russia, a large-scale study of 112 clinical *C. auris* isolates revealed markedly different antifungal resistance profiles. Nearly all the isolates (111 of 112) were fluconazole resistant, and 17% exhibited resistance to amphotericin B [48]. The prevalence of azole resistance is particularly alarming given the widespread empirical use of fluconazole, especially in intensive care settings. The high resistance rates suggest that clade I strains, which were dominant in this cohort, likely acquired multiple resistance mechanisms, possibly through antifungal pressure in nosocomial environments. Furthermore, 3.6% of the isolates were also resistant to flucytosine, and a small fraction demonstrated reduced susceptibility to echinocandins. This multidrug-resistant phenotype mirrors global observations where clade I strains are associated with hospital outbreaks and treatment failure. The concurrent phospholipase activity found in many of these isolates also implies that resistance may be coupled with high virulence, exacerbating clinical outcomes [48].

The Italian experience further underscores the challenges of treating *C. auris* infections with standard antifungal regimens. A case of candidaemia in a critically ill ICU patient in southern Italy revealed high clonality between isolates from two different patients, suggesting nosocomial transmission [49]. Both strains belong to clade I and demonstrated elevated MICs of fluconazole and borderline susceptibility to amphotericin B, which is consistent with the tentative CDC breakpoints. Interestingly, despite the administration of caspofungin during empiric therapy, the patient’s condition deteriorated rapidly, suggesting that susceptibility to echinocandin may not always translate to clinical success. Environmental surveillance in the ICU also detected *C. auris* DNA on nearly half of high-touch surfaces, emphasizing the environmental persistence and potential for reinfection. These findings support the inclusion of antifungal resistance screening in infection control measures and suggest a need for environmental decontamination strategies to accompany clinical treatment.

A significant factor complicating antifungal therapy in *C. auris* infections is the variability in susceptibility testing methods and interpretations. Arendrup et al. [50] compared CLSI, EUCAST, and commercial strip-based methods (Etest, MTS) and reported significant discrepancies in the categorisation of amphotericin B resistance. These differences in interpretation of susceptibility may lead to either overestimation or underestimation of resistance rates, affecting treatment outcomes [51]. The biofilm formation and hydrolytic enzyme activity of resistant strains may further complicate their eradication and treatment. Testing method discrepancies must also be resolved to ensure accurate resistance detection. Overall, integrated surveillance, standardized testing, and molecular typing are indispensable for guiding therapy and outbreak control strategies [52].

The number of studies from Türkiye reporting *C. auris* infections has increased in recent years [16, 34, 53–56]. However, biocide susceptibility has been evaluated in only two studies [16, 34]. Our current study represents Turkish isolates; however, it has several limitations that should be acknowledged. First, all 47 isolates analysed belonged exclusively to Clade 1, which restricts the generalisability of the findings across other *C. auris* clades. However, Clade 1 remains the most globally prevalent and clinically relevant clade, particularly in outbreak scenarios, and our findings contribute to filling the gap in region-specific data from Turkey, where such clade-based susceptibility data are limited. The phenotypic diversity observed in virulence and susceptibility traits may differ between isolates from Clades II–V and the recently described African genotype. Second, the interpretations of reduced susceptibility to biocides were based on epidemiological cut-off values (ECOFFs) extrapolated from *C. albicans*, as standardized biocide breakpoints for *C. auris* are currently lacking. This may lead to potential misclassification of resistance. However, it enables standardized comparisons and has been adopted in other peer-reviewed studies in the absence of formal CLSI or EUCAST guidelines for biocides. Third, virulence assessment relies primarily on in vitro assays, which may not fully replicate host‒pathogen interactions in vivo. Additionally, all analyses were based on phenotypic methods without integrating genomic resistance or virulence markers, which limits mechanistic insight into the observed correlations. Nonetheless, standardized phenotypic assays such as esterase, phospholipase, and biofilm production remain valuable and widely accepted surrogates for virulence screening in initial pathogenesis studies. Future research integrating in vivo models and genomic analyses is warranted and is planned as a follow-up to this work. Finally, although cross-resistance between antifungal and biocide MICs was explored using nonparametric methods, the study design was observational and hypothesis-generating; causal relationships cannot be inferred. While our correlation analysis revealed statistically significant associations between antifungal/reduced susceptibility to biocides and virulence factors, the design is exploratory and does not support causal inference. We hope that by openly discussing these limitations, we have provided sufficient transparency while reinforcing the relevance and applicability of our findings for understanding the evolving threat of Clade 1 *C. auris* in clinical settings.

## Conclusions

*Candidozyma auris* has 6 genotypes, including the last identified Bangladesh lineage [37]. Genotypes may differ in their susceptibility to both antifungals and biocides, as well as in their virulence characteristics [57–59]. This study provides a comprehensive phenotypic characterization of 47 *Candidozyma auris* Clade 1 clinical isolates from Türkiye, highlighting their antifungal and biocide susceptibility profiles alongside virulence factor expression. High resistance rates to fluconazole and variable responses to biocides such as triclosan and benzalkonium chloride were observed. All isolates demonstrated biofilm and secreted proteinase activity, while esterase and caseinase expression showed inter-isolate variability.

Statistically significant correlations between antifungal MICs (particularly amphotericin B) and certain biocide MICs and virulence markers suggest possible phenotypic convergence, although these findings do not imply shared resistance mechanisms. Importantly, resistance and pathogenicity are driven by distinct biological pathways and should not be conflated.

These findings underscore the need for integrated surveillance of antifungal and disinfectant susceptibility, particularly for outbreak-prone pathogens like *C. auris*. Future studies should incorporate genomic and transcriptomic approaches to validate the phenotypic patterns observed and to develop standardized testing frameworks for hospital-use biocides against fungal pathogens.

## Supplementary Information

Supplementary Material 1.

## Data Availability

No datasets were generated or analysed during the current study.
